# WHO competency framework for health authorities and institutions to manage infodemics: its development and features

**DOI:** 10.1186/s12960-022-00733-0

**Published:** 2022-05-07

**Authors:** Sara Rubinelli, Tina D. Purnat, Elisabeth Wihelm, Denise Traicoff, Apophia Namageyo-Funa, Angus Thomson, Claire Wardle, Jaya Lamichhane, Sylvie Briand, Tim Nguyen

**Affiliations:** 1 Department of Health Sciences and Health Policy, University of Lucerne and Swiss Paraplegic Research, Frohburgstrasse 3, 6002 Lucerne, Switzerland; 2 Unit for High Impact Events Preparedness, Department of Epidemic and Pandemic Preparedness and Prevention, Emergency Preparedness Programme, World Health Organization, Ave Appia 21, 1202 Geneva, Switzerland; 3 Demand for Immunization Team, Global Immunization Division, US Centers for Disease Control and Prevention, 1600 Clifton Rd NE, Atlanta, USA; 4 Workforce Development Team, Global Immunization Division, US Centers for Disease Control and Prevention, 1600 Clifton Rd NE, Atlanta, USA; 5 Demand for Immunization, Health Section, UNICEF, Plaza, NY 3 UN USA; 6 School of Public Health, Brown University, 121 S Main St, Providence, RI 02903 USA; 7 Department of Epidemic and Pandemic Preparedness and Prevention, Emergency Preparedness Programme, World Health Organization, Ave Appia 21, 1202 Geneva, Switzerland

**Keywords:** Infodemic, Competencies, Workforce

## Abstract

**Background:**

In April 2020, the World Health Organization (WHO) Information Network for Epidemics produced an agenda for managing the COVID-19 infodemic. “Infodemic” refers to the overabundance of information—including mis- and disinformation. In this agenda it was pointed out the need to create a competency framework for infodemic management (IM). This framework was released by WHO on 20th September 2021. This paper presents the WHO framework for IM by highlighting the different investigative steps behind its development.

**Methods:**

The framework was built through three steps. Step 1 included the preparatory work following the guidelines in the Guide to writing Competency Framework for WHO Academy courses. Step 2 was based on a qualitative study with participants (*N* = 25), identified worldwide on the basis of their academic background in relevant fields of IM or of their professional experience in IM activities at the institutional level. The interviews were conducted online between December 2020 and January 2021, they were video-recorded and analyzed using thematic analysis. In Step 3, two stakeholder panels were conducted to revise the framework.

**Results:**

The competency framework contains four primary domains, each of which comprised main activities, related tasks, and knowledge and skills. It identifies competencies to manage and monitor infodemics, to design, conduct and evaluate appropriate interventions, as well as to strengthen health systems. Its main purpose is to assist institutions in reinforcing their IM capacities and implementing effective IM processes and actions according to their individual contexts and resources.

**Conclusion:**

The competency framework is not intended to be a regulatory document nor a training curriculum. As a WHO initiative, it serves as a reference tool to be applied according to local priorities and needs within the different countries. This framework can assist institutions in strengthening IM capacity by hiring, staff development, and human resources planning.

## Background

COVID-19 is the first pandemic in history in which different technologies and social media have been at the core of communication aimed at providing information and keeping people connected [1–5]. The same technology, however, has amplified infodemics—an overabundance of information—through different online and offline communication channels, particularly the dissemination of mis- and dis-information. Misinformation is false or misleading information, but the person who disseminates it believes to be true. Disinformation is false or misleading information, and the person who disseminates it knows it is of low-quality [6–8]. Suboptimal information undermines the public health response to the pandemic, negatively impacting people’s physical and mental health and hampering the responses of countries to the pandemic [9, 10]. Mis- and disinformation can polarize the public debate and promote hate speech, thus threatening human rights and social cohesion [11, 12]. Infodemics during COVID-19 has pointed to the need to identify existing instruments and to develop new frameworks and tools to manage it.

On February 15, 2020, the WHO Director-General, Tedros Adhanom Ghebreyesus, warned the world of the threat of an infodemic accompanying the pandemic [13]. In April 2020, the UN Secretary-General launched the United Nations Communications Response Initiative to combat the spread of mis- and disinformation [14], and on May 11, 2020, it issued a “Guidance note on addressing and countering COVID-19 related hate speech” [15].

Between June and October of 2020, the WHO Information Network for Epidemics (EPI-WIN) organized a global online technical conference to develop a public health research agenda for infodemic management [16–18]. This event strengthened the foundations of infodemiology—the science of mitigating public health problems resulting from an infodemic [19, 20]. Through the identification of examples, practices, and tools, the conference comprehensively defined how to establish a community of experts to guide research and implement long-term and sustainable practices of IM. Prominent in this discussion was the need for health institutions and organizations to develop expertise in IM to promote resilience to the infodemic in individuals and communities.

To address this need, WHO, in partnership with the US Centers for Disease Control and Prevention (US CDC), conducted a multistep investigative process to collect relevant information and developed a framework with a set of actions needed for IM and the tasks, skills, and knowledge required for implementation. The competency framework was released by WHO on 20th September 2021 [21].

This paper describes how the competency framework for IM was developed and explains the contents in detail.

## Methods

The 2021 WHO *Competency Framework for Infodemic Management* was developed in five main steps that link together conceptual work (steps one and two) and participatory research with the relevant stakeholders (steps three, four, and five).

First, the overall structure of the framework was built following the guidelines for competency frameworks by the WHO Academy, a WHO training institution that focuses on lifelong training within the health sector [22]. Specifically, adopting the conceptualization of WHO Academy, the competencies were organized into the following categories: domains, activities, tasks, knowledge, and skills. The term “domains” is used for the headings that highlight a group of related competencies (e.g., the domain “detect and intervene”, which groups together the competencies needed to identify mis- and disinformation and build interventions to promote resilience in individuals and communities). “Activities” refers to the core functions of IM work with the characteristics of being trainable and, through the performance of tasks, measurable (e.g., to counter mis- and disinformation—that is, to offer corrections in a timely manner). The term “tasks” refers to the observable units of work within an activity (e.g., the task of “working in partnership with other institutions to identify mis- and disinformation rapidly”). “Knowledge” and “skills” refer to the informational basis needed to perform a certain task as well as the specific abilities that are required for such (e.g., knowledge of approaches and methods for fact-checking and the related skills).

Second, the domains and activities were identified by framing infodemic management within an infodemiologic perspective. Infodemiology conceptualizes five workstreams in the epi curve of an infodemic response analogous to the epidemic response [23]. These workstreams are at the core of the domains and define the related activities.

Third, the specific tasks, knowledge, and skills required for the performance of each activity were identified through a qualitative study with key participants identified purposively. Specifically, the participants (*n* = 26) were interviewed based on their academic background in the field of IM (*n* = 10) or their professional experience in IM activities at the institutional level, governmental public health agencies, or public health organization and institutions (*n* = 16). They were active in the following countries or regions: Africa (*n* = 3), Belgium (*n* = 1), Canada (*n* = 2), China (*n* = 1), Finland (*n* = 1), Italy (*n* = 2), Malta (*n* = 1), Pakistan (*n* = 1), Sweden (*n* = 1), Switzerland (*n* = 1), Thailand (*n* = 1), UK (*n* = 3) and US (*n* = 8). The participants had interdisciplinary expertise in the following fields: informatics, health behavior change, health communication, health economics, health education, health literacy, health policy, public health, scientific journalism, and social media.

The interview grids focused on the following topics:

- current IM processes within institutions (strengths and limitations, gaps, and needs);
- specific theories, models, strategies, and tools for IM used within institutions; and
- key disciplines for competence development in IM.

The full interview grids are available in Annex 1.

The interviews were conducted via videoconference between December 2020 and January 2021; they were video-recorded and transcribed verbatim. The transcripts were then analyzed using inductive thematic analysis [24].

Fourth, the participants in the qualitative study highlighted different tasks, fields, theories, models, strategies, practices, and processes that are important for IM. All these findings were clustered under standardized categories and then inserted in the draft framework under the specific domains and activities.

Fifth, the draft framework was presented for discussion and revision during two stakeholder panels held on January 26, 2021 and February 2, 2021 via videoconference. The panels took place with a majority of the participants in the qualitative study (*n* = 14), academics (*n* = 5) and practitioners (*n* = 11), some additional academics (*n* = 2), and members of the WHO core team for IM (*n* = 6). Overall, 21 people took part in the first panel, and 17 in the second panel. The panelists were mainly asked to express their views on whether the framework covers all the main IM competencies and to identify aspects that were unclear, were missing, or would require different wording. The framework was revised according to the results of the two panels.

## Results

### Domains and activities

**Fig. 1:**
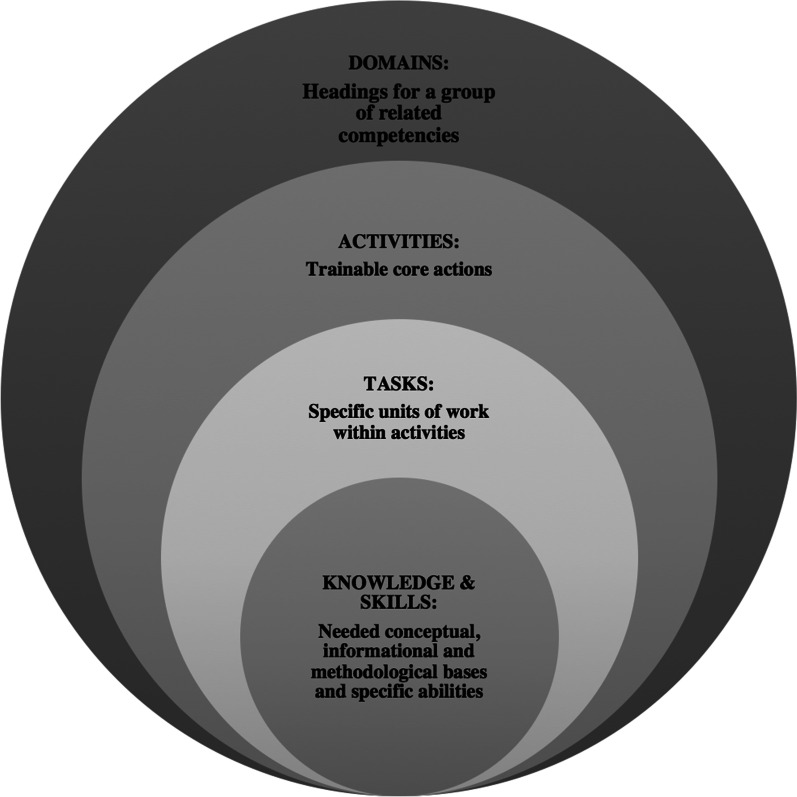
Components of the competency framework

Following step two in the methodological section above, the IM competencies are framed under four main concepts that mirror the management of epidemics [18]. Specifically, these concepts derived from WHO approach that links IM to the epidemiological concepts of surveillance, virus, disease, and interventions (Fig. 1).

**Fig. 2:**
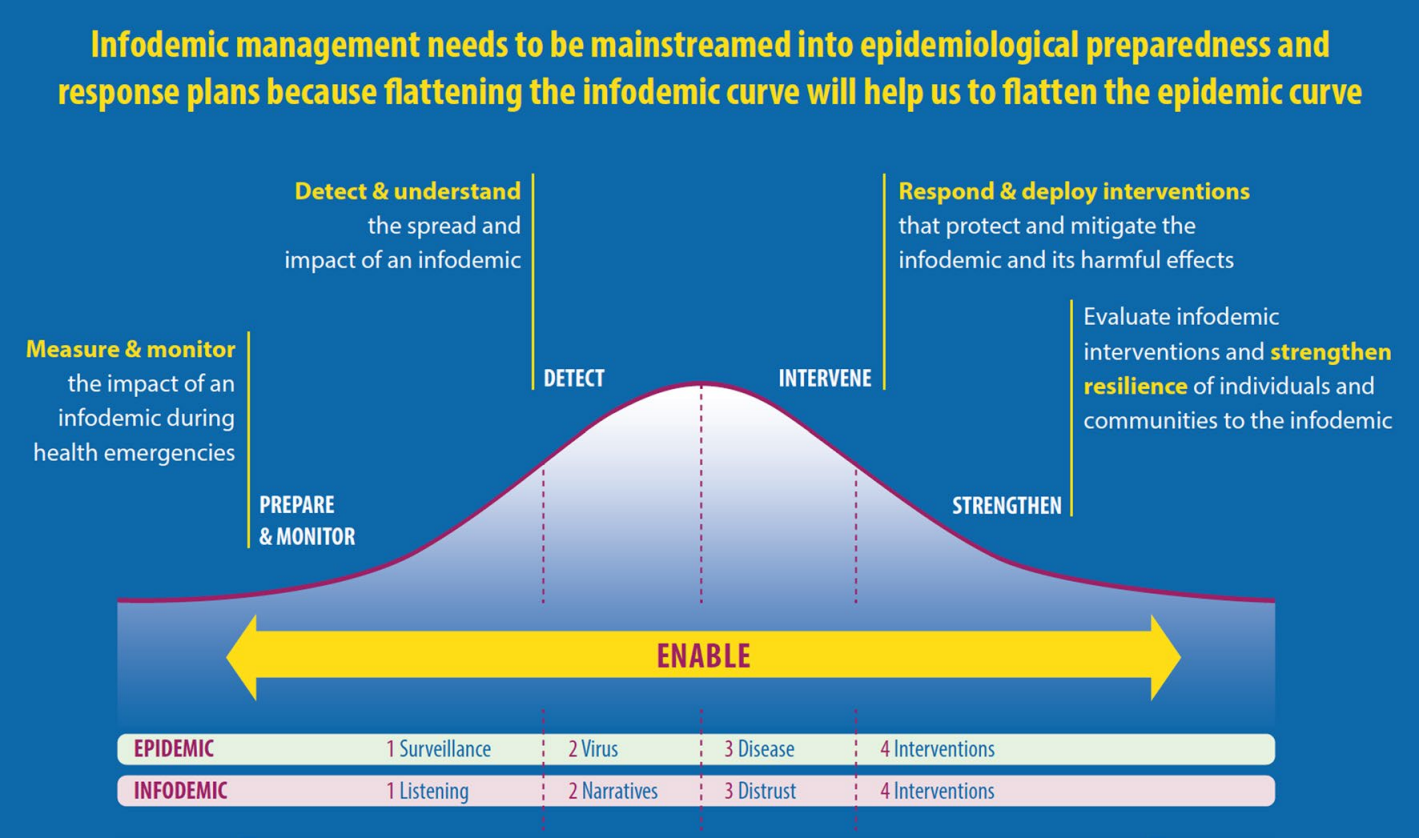
The five workstreams in the epi curve of an infodemic response analogous to the epidemic response

These concepts are:

Social listening: It refers to the systematic collection, analysis, and interpretation of people's questions, concerns, information voids, and narratives, includinf mis/disinformation that are exchanged through off-and online communication channels. Social listening insights are analysed with insights from other kinds of epidemiological, behavioral, informaton ecosystem and health information system insights, through integrated analysis to diagnose barriers and enablers of people's adherence to health guidance and enactment of health behaviors.
Narratives: It refers to both the identification of narratives that refer to a theme of conversaiton online, including  mis/disinformation and to the design, dissemination, and evaluation of narratives that can strengthen resilience to infodemics.
Distrust: It refers to both the importance of trust in health authorities, health response, and the need to identify distrust of recommendations and to promote and evaluate the impact of interventions to build institutional collaboration and engagement to protect people and lower the risks of the disease.
Interventions: In the field of IM, this refers to actions aimed at flattening the epi curve and building resilience to infodemics among populations (Fig. 2).

The main IM concepts are then operationalized in five streams that provide an overview of the main activities needed to flatten the epi curve:

- Workstream 1. *Measure and monitor the impact of infodemics during health emergencies*. The application of standardized metrics and tools are needed to track the evolution of infodemics among individuals, communities, societies, and health systems—in both digital and the physical information environments. This workstream is based in the IM domain of the competency framework called “prepare and monitor” (Fig. 1) and its related activities (Table 1).

**Table 1:** Domains of the IM competency framework and related activities

Domain (1)	1. Infodemic management
*Competency statement*	Infodemic managers apply IM and the science of infodemiology to public health policies, programs and practice
Activity	1.1 Coordinate, facilitate and strengthen
*Objective of the activity*	Promote and facilitate implementation of IM within institutions
Domain (2)	2. Prepare and monitor
*Competency statement*	Infodemic managers use effective tools to listen to target audiences and have the skills to design and share appropriate information
Activity	2.1 Listen
*Objective of the activity*	Listen, identify and understand population gaps, needs, behaviors and their determinants to develop more responsive health programs
Activity	2.2 Inform
*Objective of the activity*	Proactively share accurate, credible and appropriate information to target audiences to increase awareness, to build and strengthen health literacy, and to promote healthy behaviors on health issues
Domain (3)	3. Detect and intervene
*Competency statement*	Infodemic managers design, implement and evaluate interventions to promote resilience to misinformation and empower individuals and communities in exercising their right to access quality health information
Activity	3.1 Intervene
*Objective*	Empower individuals and communities to mitigate harm of mis/disinformation
Activity	3.2 Counter
*Objective*	Offer corrections in a timely way that match how the mis/disinformation is spread
Activity	3.3 Monitor
*Objective*	Measure the impact of interventions and countering/correction strategies
Activity	3.4 Support
*Objective*	Support individuals’ and communities’ resilience against mis/disinformation
Domain (4)	4. Strengthen
*Competency framework*	Infodemic managers strengthen health systems to ensure healthier populations through a better IM in health emergencies and in regular contexts
Activity	4.1 Prepare
*Objective*	Ensure that data-based insights and lessons learned from interventions are applied to prepare health systems with planning, processes and policies for IM
Activity	4.2 Ongoing monitoring and strategy refinement
*Objective*	Implement regular and nimble feedback and a refinement process to adapt to the changing needs of the target populations
Activity	4.3 Building capacity
*Objective*	Build IM capacity within institutions
- Workstream 2. *Detect and understand the spread and impact of infodemics*. A common approach among institutions is needed to understand how information and mis- and disinformation is spread and how it affects online and offline behavior among different populations. This workstream is based on the IM domain of the competency framework called “detect” (Fig. 1) and its related activities (Table 1).
- Workstream 3. *Respond and deploy interventions that mitigate and protect against the infodemic and its harmful effects*. An evidence base is needed to identify interventions that are effective in different contexts and for different types of acute health events. This workstream is based on the IM domain of the competency framework called “intervene” (Fig. 1) and its related activities (Table 1).
- Workstream 4. *Evaluate infodemic interventions and strengthen the resilience of individuals and communities to infodemics*. Common evaluation frames are needed to improve the development of interventions and programmatic responses to infodemics. This workstream is based on the IM domain of the competency framework called “strengthen” (Fig. 1) and its related activities (Table 1).
- Workstream 5. *Enable the development, adaptation, and application of tools for the management of infodemics*. There is a need to enhance the transferability of lessons learned from IM and evidence-based interventions between contexts, countries and infodemics. This workstream is based on the IM domain of the competency framework called “infodemic management” and its related activities (Table 1).

Table 1 lists the domains of the IM competency frameworks and their related activities. Each domain is briefly explained with a competency statement that highlights the core aspects of the competencies that fall under that specific domain. Each activity is presented with its main objective, which explains the activity’s expected result.

### Tasks

Participants in the qualitative interviews highlighted some main guiding principles on what IM managers specifically should do to enhance the performance of each activity identified in the prior step. They focused on what could potentially affect one or more phases of managing the infodemic curve. These principles highlighted the specific tasks and their classification within the four IM domains. Those principles that emerged from several participants are reported below and are illustrated through sample excerpts from the different interviews.

1. *Institutional capacity*. Health institutions’ awareness of IM and the strengthening of their IM resources is key:
  “Institutional capacity requires finding human resources and having, among other things, a legal framework. Here, governments have to partner among themselves and show awareness of the importance of IM”. (Participant H)
2. *Ongoing education*. IM is a process—not a single phase—that results in regular updating to educate communities.
  “Let the public know when relevant new information about the pandemic becomes available and explain how new information may change pandemic guidelines.” (Participant D)
  This ongoing process is fundamental to avoid an information vacuum:
  “Remember that whenever there is an information vacuum, people will try to fill in this vacuum. This has led to lot of speculation.” (Participant E)
3. *Targeting communication*. Messages have to be relevant to people according to where they stand, their knowledge and their health literacy.
  “We must consider what form of communication people need (written, audio, visual, and so forth). We have to decide the best speaker for a certain message: a press officer, a good storyteller, an expert in scientific findings. We need influencers, but it also important to have as testimonial normal people.” (Participant B)
  This same participant also stated the following:
  “The language of our messages is fundamental, it has to be context-dependent and clear, otherwise people won’t relate to what we say and, even worse, they won’t understand what we say. (Participant S)
  This implies careful attention to cultural and infrastructural factors.
  “It is clear that different audiences have different needs and perspectives in an emergency. So, we must assess all these things in terms of people’s experience and perceived level of risk. Also, physical access to communication varies due to communication channel availability, infrastructure, personal choice, social norms, and the economic situation”. (Participant C)
4. *Interactivity*. There must be channels where audience members can ask questions and receive answers in real time. Specifically:
  “These can be online forums, telephone hotlines, community meetings, but it is also fundamental to educate health care providers and relevant others to answer questions about the pandemic.” (Participant F)
  Feedback was found to be essential to refining the communication strategy:
  “Solicit public feedback. Audience feedback is very valuable, as it can help you adapt your messages to different audience segments. Also, you can refine your messages so that you increase acceptability.” (Participant G)
5. *Inclusion.* IM needs specific strategies to address those people who, because of economic, cultural, or historical factors, have a different lived experience of health institutions, as well as access to information.
  “Building relationships with marginalized people is essential. Everybody suddenly is important!”. (Participant P)
  Within inclusion, access and equity are two main principles to support populations:
  “Accessibility is the key word. We need to make sure that everybody receives and understands communication.”. (Participant W)
  and:
  “You also have to consider that distrust is being driven by a growing sense of inequity and unfairness in the system. It is important to consider everybody and not to exacerbate the feeling that institutions only serve the interests of the few over everyone”. (Participant R)
6. *Quality of information*. Freedom of speech is an asset of democracy; however, suboptimal information can negatively impact health decision-making. Thus, there needs to be an established norm regarding information quality:
  “Freedom of speech for sure, but it should also have limitations. You can have it until it does not harm. There are many legal aspects linked to the control of information. Acting on the basis of what we monitor is not easy. What do you do? Do you block the accounts of people? This is not feasible in a complex system. People have to know how to distinguish between information of different quality.” (Participant Z)
7. *What is known/unknown.* Health institutions have to be quite honest and transparent about the current state of evidence as well as its strengths and limitations. This is also because a lack of clarity or inconsistency leads to a lack of trust:
  “We are vulnerable to losing trust. When there is no data, we have to be very honest and transparent about that.” (Participant A)
8. *Scientific literacy*. Since the discussion of health threats entered the scientific domain, and people on average do not have the competencies to understand scientific thinking, there is a need to strengthen scientific literacy in the different populations. This is particularly important when individuals, communities, or organizations appear to be in disagreement among themselves:
  “People disagree over scientific issues, but, of course, if scientists do not agree, this creates confusion, and then people start to believe what they prefer and what is closer to them.” (Participant I)
  Moreover:
  “Science cannot provide immediate answers to a new phenomenon. We need to deal with uncertainty (with HIV, it was the same thing). At the beginning, people have so many questions, and often there is a lack of good information.” (Participant L)
  Disinformation can be prompted by a perceived inconsistency, and this has to be carefully addressed through clear communication:
  “Inconsistent messages increase anxiety and quickly undermine expert advice and credibility. In reality, you cannot control what someone else says, but, by fully and clearly explaining your messages and their reasoning, your audiences will be less likely to doubt you.” (Participant M)
  Scientific literacy should be considered in the context of health literacy, especially critical health literacy:
  “This is not only about being able to read and understand health information. People have to grow some basic understanding of how science functions in order to appraise at least the difference between what is a personal opinion and what is scientific evidence”. (Participant I)
9. *Partnership*: IM requires collaboration, partnership, and coordination.
  “There is no such person who can have all of the skills. We really need to understand what happens at the community level and whether or not communities are following. This is complex to analyze and requires different expertise.” (Participant H)
  In general, partnership is most valuable when it avoids duplicating efforts and strengthening interventions from countries that lack resources:
  “Networking is easy, but, unfortunately, many of us are doing the same things in different contexts and thus reduplicating things”. (Participant L)
  The participants detailed specific tasks for IM, which were then attributed to each domain and related activities of the competency framework. Table 2 presents a summary of the main tasks for each activity of the IM competency framework. The full list of tasks is available in the actual WHO competency framework. [21]

**Table 2:** IM tasks according to the related activities

Domain (1)	1. Infodemic management
Activity	1.1 Coordinate, facilitate and strengthen
Main tasks:	• Develop or adopt a taxonomy of classifications for mis/disinformation as a reference framework for IM • Promote and ensure coordination among the different domains and tasks of IM • Develop partnerships with organizations that are active in IM • Promote ethical conduct in IM to avoid the spread and propagation of harmful health information, as well as unintended harm from all actions
Domain (2)	2. Prepare and monitor
Activity	2.1 Listen
Main tasks:	• Analyze and evaluate individuals’ behaviors, focusing on personal, social and environmental determinants • Identify people’s topics of interest • Detect information deficits and open questions in the offline and online populations • Identify, analyze and evaluate the evidence-basis of the main narratives and claims over health issues circulating in the population
Activity	2.2 Inform
Main tasks:	• Develop and tailor messages for different populations, utilizing appropriate communication strategies, communication media and channels • Pretest messages among target populations and in different media • Measure the effectiveness of messages in time and media • Partner with medical associations, nongovernmental organizations, traditional and social media, and tech companies to target different stakeholders in the health system • Promote credibility and trust in health authorities and service delivery
Domain (3)	3. Detect and intervene
Activity	3.1 Intervene
Main tasks:	• Define the objective of the single intervention or of the multiple interventions, and the target populations • Identify barriers to and facilitators of the planned objective in the target population • Define a model of change and clarify processes which will be used to assess the efficacy of the intervention • Define the various levels the intervention covers, from policy and health system to community and individual levels • Produce the interventions and implement them
Activity	3.2 Counter
Main tasks:	• Build or strengthen reporting tools and processes to identify and analyze mis/disinformation • Track mis/disinformation, check facts and trends over time • Work in partnership with stakeholders to identify and act on mis/disinformation rapidly
Activity	3.3 Monitor
Main tasks:	• Collect and collate data related to interventions and messages • Estimate the impact of the interventions • Transfer the findings of interventions to improve mis/disinformation correction and management
Activity	3.4 Support
Main tasks:	• Design, implement and evaluate interventions to build and strengthen resilience against mis/disinformation, tailored to individual communities and vulnerable populations • Measure community involvement and empowerment • Integrate measures for infodemic resilience into health system standard reporting processes
Domain (4)	4. Strengthen
Activity	4.1 Prepare
Main tasks	• Promote building, revision and adoption of policies for IM • Embed IM modules and indicators in all relevant aspects of the public health response • Support and promote interdisciplinarity in institutions’ IM
Activity	4.2 Ongoing monitoring and strategy refinement
Main tasks	• Identify and address gaps in IM program design and service delivery • Use implementation research evidence in program improvement and policy development • Document IM processes, analyses and outputs for future use • Promote shared interventions and approaches between countries, including the assessment of factors affecting the transferability of interventions
Activity	4.3 Building capacity
Main tasks	• Assess IM training needs within the institution • Set organizational training objectives and create training action plans • Define and plan for internally provided or outsourced training • Implement training initiatives • Evaluate and revise training • Integrate infodemic training within the main processes and services for employees of the institution

### Knowledge and skills

The practice of IM is interdisciplinary and requires coordinated expertise from different disciplines. Participants in the interviews highlighted the main disciplines needed as the content basis for IM and the related skills. Table 3 lists the main IM disciplines in alphabetical order as well as the main IM skills, contextualizing them according to the domains and activities for which they are most needed.

**Table 3:** Disciplines and skills of IM according to the domains and activities

Disciplines involved in IM	Specific skills	IM domains where the specific skills are needed
Advertising Advocacy Argumentation theory Behavioral sciences Cognitive science Communication sciences (from interpersonal to mass communication) Community engagement Complexity science Computational social science Cybersecurity Design Digital health Education and pedagogical sciences Ethics Health campaigns Health communication Health economics Health informatics Health literacy Health service research Health system research Knowledge translation Knowledge dissemination Implementation science Infodemiology and IM (theories, methods, tools, strategies and processes) Institutional development Law Media and Journalism Media literacy Narratology and the rhetoric of narratives Persuasion research Public health (history of public health and best practice) Organizational management Quantitative and qualitative research methods Risk communication Science literacy Scientific journalism Social listening and social media monitoring tools (Social) marketing Social inequalities and health inequity Study design Team communication User experience design (UXD)	• Strengthen and develop all main IM processes within institutions • Identify and apply standards for ethical conduct in IM • Build a network of partners for coordinated IM	Domain 1 Infodemic management
	• Identify mis/disinformation • Utilize research methods, social listening and social media monitoring tools and methods to collect data (online and offline) on an infodemic • Identify targets for IM interventions	Domain 2 Prepare and monitor 2.1. Listen
	• Tailor health communication and dissemination of health information • Pretest messages for relevance, readability, comprehension and potential impact • Maintain, promote and build trust in health institutions • Communicate with the media • Empower spokespersons to speak on behalf of institutions	Domain 2 Prepare and monitor 2.2. Inform
	• Develop and implement interventions that address individual, community, cultural and societal-level factors affecting trust and resilience to misinformation	Domain 3 Detect and intervene 3.1. Intervene
	• Develop and utilize standard operating procedures to collect, analyze and correct misinformation on various levels • Build and strengthen coordinated work with partner organizations and stakeholders to act on mis/disinformation in a timely way	Domain 3 Detect and intervene 3.2. Counter
	• Design and conduct impact studies • Reflect on the results of interventions to refine overall institutional strategies against infodemics	Domain 3 Detect and intervene 3.3. Monitor
	• Use frameworks and research methods to build and evaluate interventions to strengthen individuals’ and communities’ resilience against mis/disinformation • Use theories, frameworks and strategies of communication to build or reinforce trust in institutions	Domain 3 Detect and intervene 3.4. Support
	• Synthesize and present existing evidence and guidance from IM findings for specific country contexts • Apply principles and tools of knowledge translation from IM findings to empower and reinforce health systems in IM • Promote inter-organizational work and collaboration	Domain 4 Strengthen 4.1. Prepare
	• Identify strengths and limitations in institutions’ IM programs and procedures • Translate the findings from IM interventions and best practices to strengthen institutions’ strategies • Collect, synthesize and transfer the findings from partners or other relevant institutions	Domain 4 4.2 Ongoing monitoring and strategy refinement
	• Identify relevant topics, needs and gaps within institutions for IM training • Build institutional relationships with relevant stakeholders (from professional categories to the mass media) • Use the theories, methods and principles of professional learning to design, implement and evaluate training in infodemiology and IM within the institution	Domain 4 4.3 Building capacity

## Discussion

### Recommendations for implementation

The WHO competency framework for IM is a reference document that can be used by health institutions and health organizations for two main purposes: (1) to identify their competence needs and (2) to plan, organize, and reinforce their IM taskforce. It highlights the main actions to be carried out by IM managers to provide a proper response to infodemics. For each of these actions, specific competencies are needed to activate the IM strategy in a comprehensive way. The framework extensively presents the main competencies for IM that can then be selected by organizations and institutions in their own country according to their needs, the infodemic scenarios they face, their resources and cultural norms. Thus, this framework is not a regulatory document, but, as a reference tool, it should be applied locally and according to the specific characteristics of nations and their organizations. Some of the competencies outlined may not be relevant for some contexts depending on certain factors, such as capacity and resources at disposal.

In light of this, the framework can support the identification of existing competencies and those that have to be fostered going forward. It may also facilitate the development of indicators for evaluating institutional and staff performance in IM, including the modification of job descriptions, identification of required training plans, and development of supervisory guidelines. With reference to staff performance and development, the framework provides a level of detail that can then be broken down into finer levels of detail to uncover specific needs in staff development and training at individual organizations.

In addition to informing workforce planning, the IM framework can be used for the design, organization, or reorganization of work processes within institutions and organizations. Process redesign could include identification of additional tools and resources that workers might need in order to successfully complete their IM tasks.

Overall, it is clear that IM is a multidisciplinary endeavor and that, whatever the approach of the single institutions and organizations, it should be as extensive as possible and consider all relevant domains. Institutional collaboration and cooperation are here essential; indeed, IM benefits from joining resources to share expertise, practices and resources, and learn from those. Also, it benefits creating networks of management that cover all tasks, without duplicating actions, in specific more or less broad geographical areas and contexts.

### Outlining a future practice and research agenda based on the competency framework for infodemic management

The competencies and tasks in the framework can assist health authorities in implementing the main findings from the literature [25–27]. At the same time, health authorities can enrich the current findings with new data derived from their work in the field. Infodemic management is a nascent field, and will therefore benefit greatly from evaluation research.

There remains a major gap between research and practice in infodemiology. A significant proportion of evidence-based tools and guidelines generated by academic disciplines have not yet been used systematically. Health institutions and organizations must work together with researchers to identify what works, what can be improved, and what gaps exist [28, 29]. Overall, the implementation of the competency framework and the collection and analysis of related data will further enrich the research agenda of infodemic management. This calls for a global participatory effort featuring researchers and practitioners to engage with communities and promote individuals’ resilience to infodemics [29, 30].

Specifically, some main tasks that can benefit from the interaction between infodemic management research and practice, according to previous research in the field [18], include the following:

1. Develop and adopt shared classification and taxonomies of disinformation [31, 32].
2. Understand how information originates, evolves, and spreads on different platforms and channels and quantify the impact. There are many social listening tools and methods for data collection (online and offline) available [33, 34].
3. Use approaches from the behavioral and cognitive sciences, among other disciplines to understand how misinformation affects behavior in different populations, with a main focus on vulnerable populations [35–37].
4. Design, implement, and evaluate interventions at different levels of action and that address individual-, community-, cultural-, and societal-level determinants of trust and resilience against misinformation [38–40].
5. Develop regulatory and ethical principles to mitigate the spread of harmful health information at different levels of society [41, 42].
6. Strengthen infodemic management capacities in health organizations and institutions by building and reinforcing related processes and empowering interdisciplinary workforces [43–47].

Overall, the infodemic management of various health institutions and organizations in different countries can contribute to understanding how different populations (and sub-populations) have different information needs, use different channels, and face different barriers. Thus, interventions that are anchored in the specific fields of IM can result in primary evidence regarding how to reduce the transmission and impact of a disease in a tailored way.

A major task for WHO is now to monitor the implementation of the competency framework and to collect case-studies, and data on its validity, use by institutions and organizations and usefulness to further advance research and practice in the field. This process of monitoring will also inform revisions of the current frameworks and provide more quantitative data to complement the qualitative analysis that bases it.

## Conclusion

This paper presents the WHO competency framework for infodemic management by illustrating its development, implementation context, and applicability.

The framework shows that at the core of infodemic management there are key actions that should focus on measuring and monitoring the impact of infodemics during health emergencies, detecting and understanding the spread and impact of infodemics, and designing, deploying, and evaluating interventions that protect against infodemics.

Infodemic management is a process and not an end state; overall, it can be effective in maintaining or restoring confidence in health systems and authorities. However, to do so, this should be done continuously—not just when there is an outbreak. IM requires stable, active, and proactive efforts and appropriate infrastructures as well as specific policies. Infodemic management cannot be isolated from a more general reflection on people’s right to information and expression, which, along with the principles of autonomy and self-determination, is one of the core components of democracy. Last, infodemic management requires collaboration, cooperation, and sharing in terms of rich data on best practices and effective tools. Moreover, it should also be feasible to collaborate in this regard at the global level, specifically to support countries that might face difficulties in finding resources.

## Data Availability

The datasets analyzed during the current study are available from the corresponding author on reasonable request.

## Annex: Interview grid

### Interview guide for participants from the academy

A. Warming up
  1. Do you think that the provision of institutional health information to individuals and communities about COVID-19 was and is problematic? Why?
  2. What do you see as the strengths and limitations of institutional engagement with the public about COVID-19?
  3. Do you have specific examples in mind of optimal/suboptimal types of institutional engagement in the context of specific countries?
B. INFODEMIC
  1. Have you heard the term “infodemic” (used by WHO). If so, in what context?
  2. Infodemic refers to an abundance of health information and health mis/disinformation. Do you think that the infodemic is a problem? Why?
  3. Do you have any evidence of potential harm that the infodemic may have contributed to in your country or other specific countries?
  4. What are, in your view, the specific populations that are particularly vulnerable?
  5. Moving to infodemic management activities:
    - Do you have any specific strategies for monitoring the infodemic? Do you have any strategies for health message design and implementation? Do you have specific strategies for evaluating the impact of your institution’s health messages?
C. Institutional engagement towards health behaviour change
  1. What do you see as the main strategies for addressing and influencing health behaviour? Do you have specific strategies to address and influence the health behaviour of individuals and communities?
D. Institutional management of infodemics
  1. If you were to advise institutions to invest in resources to manage communication/infodemics:
    - what disciplines could provide the main guidance on empowering the infodemic response and increasing resilience to an overabundance of health information and health disinformation?
    - what competencies do you think would be most valuable?
    - what type of job positions would you envisage and what job profiles would need to be skilled up?
    - what training in which fields/specific topics would you recommend for optimal public health responses?
E. Final
  1. Is there any other topic/issue that you would like to address about infodemic management to strengthen institutions?

### Interview guide for participants from health institutions

A. Warming up
  1. COVID-19 is linked to an overload of information, including misinformation and disinformation (for example, fake news and conspiracy theories). Did you or are you experiencing problems with this?
  2. Can you think of any example where your institution had to deal with, or has been impacted by, disinformation? If yes, can you recall the case and what did you do to manage it? If not, do you have examples in mind outside your institution and in your country?
  3. What do you think are the past and current major challenges in engaging with the public about COVID?
  4. In terms of communication/engagement with the public, what have you learned from the past 10 months that you find valuable today?
B. Focus on the infodemic
  For WHO, “infodemic” refers to the abundance of health information and health mis- and disinformation. In order to manage infodemics, in your institution:
  1. Do you know/use measures to listen to and understand the population’s information needs?
  2. How do you design the messages for the public?
  3. Do you use/know theories or models for behaviour change? How do you engage with your community?
  4. When you see instances of mis/disinformation that can impact your community, do you have strategies and tools to correct it in a timely way?
  5. Do you have a system in place to monitor the impact of your communication and community engagement?
  6. How do you build, maintain or restore trust with your audience? How can health institutions do this?
  7. How do you strengthen community empowerment and how do you support individual and community resilience to mis/disinformation?
C. Preparedness of health institutions
  1. Before COVID-19, did your organization have any infodemic management capacity to address health issues?
  2. During COVID-19 did your institution build or reinforce a structure to deal with health communication, especially considering the risks linked to the infodemic? Did your institution increase resources to manage the infodemic? Were new people employed?
  3. How many people work on communication and information management and community engagement? Is this number enough to deal with these tasks? If not, which additional resources would you consider important, with what backgrounds and job profiles?
  4. Did existing employees receive specific training in this field? If there was any training, was this provided by people working internally or it was outsourced to external trainers?
  5. In managing the infodemic does your institution work in partnership with other institutions and organizations, or with other stakeholders (e.g., social media platforms)? If yes, is this collaboration beneficial and why?
  6. How do you deal in your institution with the fact that scientific evidence changes rather fast, that scientists/health practitioners often disagree? Does this disagreement also have an impact on your employees and colleagues?
D. Final
  1. Is there any other topic/issue that you would like to address about institutional engagement and management of the infodemic in order to strengthen institutional efforts?

## Abbreviations

EPI-WIN: WHO Information Network for Epidemics
IM: Infodemic management
US CDC: US Centers for Disease Control and Prevention
WHO: World Health Organization
