# Correction to: WHO competency framework for health authorities and institutions to manage infodemics: its development and features

**DOI:** 10.1186/s12960-022-00750-z

**Published:** 2022-06-03

**Authors:** Sara Rubinelli, Tina D. Purnat, Elisabeth Wilhelm, Denise Traicoff, Apophia Namageyo-Funa, Angus Thomson, Claire Wardle, Jaya Lamichhane, Sylvie Briand, Tim Nguyen

**Affiliations:** 1 Department of Health Sciences and Health Policy, University of Lucerne and Swiss Paraplegic Research, Frohburgstrasse 3, 6002 Lucerne, Switzerland; 2 Unit for High Impact Events Preparedness, Department of Epidemic and Pandemic Preparedness and Prevention, Emergency Preparedness Programme, World Health Organization, Ave Appia 21, 1202 Geneva, Switzerland; 3 Demand for Immunization Team, Global Immunization Division, US Centers for Disease Control and Prevention, 1600 Clifton Rd NE, Atlanta, USA; 4 Workforce Development Team, Global Immunization Division, US Centers for Disease Control and Prevention, 1600 Clifton Rd NE, Atlanta, USA; 5 Demand for Immunization, Health Section, UNICEF, Plaza, NY 3 UN USA; 6 School of Public Health, Brown University, 121 S Main St, Providence, RI 02903 USA; 7 Department of Epidemic and Pandemic Preparedness and Prevention, Emergency Preparedness Programme, World Health Organization, Ave Appia 21, 1202 Geneva, Switzerland

## Correction to: Human Resources for Health (2022) 20:35 10.1186/s12960-022-00733-0

Following publication of the original article [1], the authors identified an error in the author name of Elisabeth Wilhelm.

The incorrect author name is: Elisabeth Wihelm

The correct author name is: Elisabeth Wilhelm

The author group has been updated above and the original article [1] has been corrected.
